# BODY AND MIND: EXPLORING ASSOCIATIONS AMONG PARALLEL TRAJECTORIES OF BIOMARKERS AND DEPRESSION SCORES

**DOI:** 10.1093/geroni/igae098.0926

**Published:** 2024-12-31

**Authors:** Brian Beach, Paola Zaninotto, Eun-Jung Shim

**Affiliations:** University College London, London, England, United Kingdom; University College London, London, England, United Kingdom; Pusan National University, Busan, Republic of Korea

## Abstract

Multiple studies have found associations between levels of various blood-based biomarkers and depression, including how some biomarkers are associated with future development of depression or the worsening of depressive symptoms. The nature of such associations, however, cannot demonstrate a causal relationship between levels of biomarkers and depression. With recent advances in data collection, this study examines whether associations exist between the trajectories of biomarkers and depression scores. It draws on data from the English Longitudinal Study of Ageing, using four measurement points for depression and nine distinct biomarkers among respondents aged 50+ collected during nurse visits between 2004 and 2019. Associations in joint trajectories are assessed using a series of parallel process latent growth models, testing goodness of fit for both linear and quadratic models. Results suggest associations between blood hemoglobin and depression, with no significant associations among the other biomarkers. More specifically, intercepts and slopes demonstrate joint negative associations, while the intercept of depression is positively associated with the rate of change for hemoglobin (β=0.029; 95%CI: 0.004,0.054). When stratified by sex, the associations with blood hemoglobin remains only for women, while men display a negative association between the intercept of depression and the rate of change for HDL cholesterol (β=-0.016; 95%CI: -0.029,-0.004). Overall, results find no relationships between change in depression scores and levels of biomarkers, suggesting little diagnostic utility of these biomarkers to identify risk for worsening depression.

